# 1-[3-(Morpholin-4-yl)prop­yl]-4-(3-nitro­phen­yl)spiro­[azetidine-3,9′-xanthen]-2-one

**DOI:** 10.1107/S160053681400419X

**Published:** 2014-02-28

**Authors:** Ísmail Çelik, Mehmet Akkurt, Aliasghar Jarrahpour, Roghayeh Heiran, Namık Özdemir

**Affiliations:** a Department of Physics, Faculty of Sciences, Cumhuriyet University, 58140 Sivas, Turkey; b Department of Physics, Faculty of Sciences, Erciyes University, 38039 Kayseri, Turkey; c Department of Chemistry, College of Sciences, Shiraz University, 71454 Shiraz, Iran; d Department of Physics, Faculty of Arts and Sciences, Ondokuz Mayıs University, 55139 Samsun, Turkey

## Abstract

The β-lactam (azetidin-2-one) ring of the title compound, C_28_H_27_N_3_O_5_, is nearly planar [maximum deviation = 0.010 (1) Å] and makes dihedral angles of 75.77 (5), 52.78 (9) and 88.72 (5)°, respectively, with the benzene ring, the least-squares plane formed by the four C atoms of the morpholine ring, which adopts a chair conformation, and the xanthene ring system. In the crystal, C—H⋯O hydrogen-bond contacts connect neighbouring mol­ecules into infinite zigzag chains running parallel to the *b* axis.

## Related literature   

For general background to β-lactams, see: Arumugam *et al.* (2011 ▶); Jarrahpour *et al.* (2010 ▶); Chrysselis *et al.* (2000 ▶); Mehta *et al.* (2010 ▶); Singh (2003 ▶); Singh *et al.* (2011 ▶, 2014 ▶). For similar structures, see: Akkurt *et al.* (2008*a* ▶,*b* ▶); Yalçın *et al.* (2009 ▶); Çelik *et al.* (2009*a* ▶,*b* ▶). For geometric analysis, see: Cremer & Pople (1975 ▶); Nardelli (1995 ▶). 
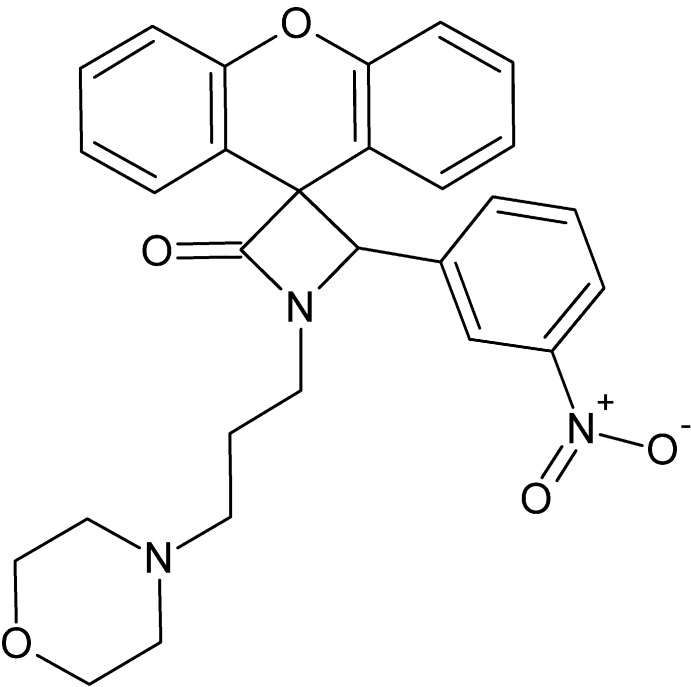


## Experimental   

### 

#### Crystal data  

C_28_H_27_N_3_O_5_
*M* *_r_* = 485.53
Monoclinic, 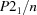

*a* = 9.2637 (6) Å
*b* = 11.4091 (5) Å
*c* = 23.3310 (15) Å
β = 96.930 (5)°
*V* = 2447.9 (2) Å^3^
*Z* = 4
Mo *K*α radiation
μ = 0.09 mm^−1^
*T* = 296 K
0.55 × 0.38 × 0.22 mm

#### Data collection  

Stoe IPDS 2 diffractometer
Absorption correction: integration (*X-RED32*; Stoe & Cie, 2002 ▶) *T*_min_ = 0.957, *T*_max_ = 0.983
19036 measured reflections
5393 independent reflections
3044 reflections with *I* > 2σ(*I*)
*R*_int_ = 0.041

#### Refinement  

*R*[*F* ^2^ > 2σ(*F* ^2^)] = 0.041
*wR*(*F* ^2^) = 0.093
*S* = 0.88
5393 reflections
325 parameters
H-atom parameters constrained
Δρ_max_ = 0.15 e Å^−3^
Δρ_min_ = −0.21 e Å^−3^

### 

Data collection: *X-AREA* (Stoe & Cie, 2002 ▶); cell refinement: *X-AREA*; data reduction: *X-RED32* (Stoe & Cie, 2002 ▶); program(s) used to solve structure: *SHELXS2013* (Sheldrick, 2008 ▶); program(s) used to refine structure: *SHELXL2013* (Sheldrick, 2008 ▶); molecular graphics: *ORTEP-3 for Windows* (Farrugia, 2012 ▶); software used to prepare material for publication: *WinGX* (Farrugia, 2012 ▶).

## Supplementary Material

Crystal structure: contains datablock(s) global, I. DOI: 10.1107/S160053681400419X/sj5392sup1.cif

Structure factors: contains datablock(s) I. DOI: 10.1107/S160053681400419X/sj5392Isup2.hkl

Supporting information file. DOI: 10.1107/S160053681400419X/sj5392Isup3.cml

CCDC reference:

Additional supporting information:  crystallographic information; 3D view; checkCIF report

## Figures and Tables

**Table 1:** Hydrogen-bond geometry (Å, °)

*D*—H⋯*A*	*D*—H	H⋯*A*	*D*⋯*A*	*D*—H⋯*A*
C17—H17⋯O1^i^	0.93	2.42	3.2423 (19)	148

Symmetry code: (i) 
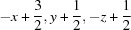
.

**Fig. 1.**
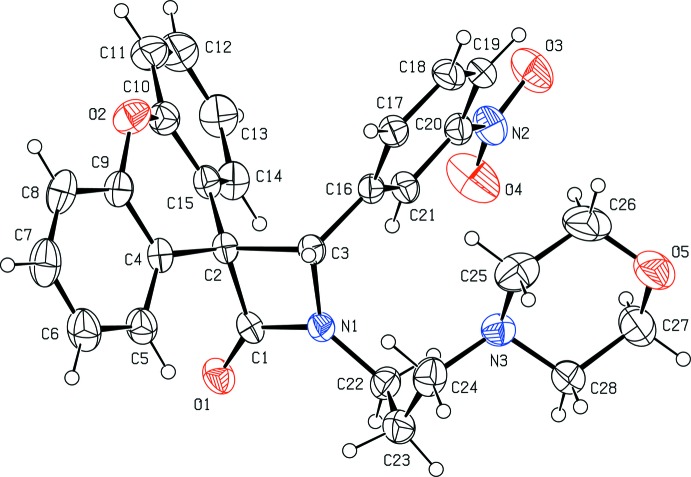
The title molecule (I) with the atom numbering scheme. Displacement ellipsoids for non-H atoms are drawn at the 30% probability level.

**Fig. 2.**
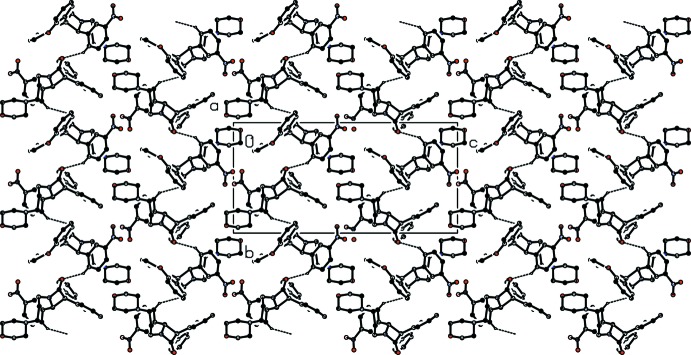
Hydrogen bonding and molecular packing of (I) viewed along the a axis. Only H atoms involved in H bonding are shown.

## supplementary crystallographic information

### 1. Comment

2-Azetidinones, commonly known as β-lactams, occupy a central place among medicinally important compounds due to their unique structure and their antibacterial activities (Arumugam *et al.*, 2011; Jarrahpour *et al.*, 2010; Singh, 2003; Singh *et al.*, 2011, 2014). β-Lactams have shown many interesting biological properties, such as acting as cholesterol absorption inhibitors, and applications as antimicrobial, antifungal, antitubercular, anticancer, anti HIV and antiparkinsonian agents (Mehta *et al.* 2010). Moreover the morpholine moiety is present in many heterocyclic compounds with hypocholesterolemic and hypolipidemic activity (Chrysselis *et al.* 2000).

The β-lactam unit in (I, Fig. 1) is nearly planar, with a maximum deviation of 0.010 (1) Å from the mean plane. Atom O1 lies almost in the β-lactam plane, with a deviation of -0.001 (1) Å. The β-lactam ring makes a dihedral angle of 75.77 (5)° with the benzene ring C16—C21.

The xanthene ring system is V-shaped, with a dihedral angle between the (C4–C9) and (C10–C15) benzene rings of 27.82 (5)°. Its central ring, C2/C4/C9/O2/C10/C15, is not planar, with puckering parameters: Q_T_ = 0.3640 (16) Å, θ = 81.6 (3)° and φ = 181.0 (3)° (Cremer & Pople, 1975). The mean plane of the xanthene ring system forms dihedral angles of 88.72 (5), 55.87 (4) and 44.54 (7)° (Nardelli, 1995), with the β-lactam ring, the benzene ring (C16–C21) and the least-squares plane formed by the four C atoms of the morpholine ring (N3/O5/C25–C28), respectively.

The bond lengths and angles in (I) are comparable with similar compounds that we have reported previously (Akkurt *et al.*, 2008*a*,*b*; Çelik *et al.*, 2009*a*,*b*; Yalçın *et al.*, 2009).

In the crystal, molecules are linked by C—H···O hydrogen contacts (Table 1) into infinite zigzag chains running parallel to [010]. Fig. 2 shows a view down the *a* axis of the crystal packing of (I).

### 2. Experimental

A mixture of *N*-(3-nitrobenzylidene)-3-morpholinopropan-1-amine (1.38 g, 5.00 mmol) and triethylamine (2.53 g, 25.00 mmol), 9*H*-xanthen-9-carboxylic acid (1.69 g, 7.50 mmol) and tosyl chloride (1.43 g, 7.50 mmol) in CH_2_Cl_2_ (25 ml) was stirred at room temperature overnight. Then it was washed with HCl 1 *M*, saturated sodium bicarbonate solution and brine, dried with Na_2_SO_4_ and the solvent was evaporated to give the crude product which was purified by column chromatography (eluent 10:1 EtOAc/EtOH) as light yellow crystalline solid (Yield 59%). mp: 424–426 K. IR (KBr, cm^-1^): 1751 (CO, β-lactam), 1342, 1527 (NO_2_). ^1^H-NMR (CDCl_3_) δ (p.p.m.): 1.94 (CH_2_—CH_2_—CH_2_–, m, 2H), 2.49 (CH_2_—CH_2_—CH_2_– and CH_2_—N morpholine ring, m, 6H), 3.18 (CH_2_—CH_2_—CH_2_–, m, 1H), 3.68 (CH_2_—O, m, 4H), 3.94 (CH_2_– CH_2_—CH_2_–, m, 1H), 4.72 (H-4, s, 1H), 5.43 (H-3, d, J = 4.4 Hz, 1H), 6.79- 7.94 (ArH, m, 12H). ^13^C-NMR (CDCl_3_) δ (p.p.m.): 24.9 (CH_2_—CH_2_—CH_2_–), 39.9 (CH_2_—CH_2_—CH_2_–), 53.5 (CH_2_—N morpholine ring), 56.0 (CH_2_—CH_2_—CH_2_–), 65.2 (C-4), 66.6 (CH_2_—O morpholine ring), 73.4 (C-3), 116.4, 116.8, 117.1, 121.2, 121.3, 122.9, 123.0, 124.4, 125.1, 127.8, 129.3, 129.4, 129.4, 132.6, 136.9, 147.9, 151.9, 152.1 (aromatic carbons), 168.1 (CO, β-lactam). GC–MS m/*z* = 485 [*M*+]. Anal. calcd for C_28_H_27_N_3_O_5_: C 69.26, H 5.61, N 8.65%. Found: C 69.30, H 5.69, N 8.67%.

### 3. Refinement

H atoms were positioned geometrically and were refined using a riding model with *U*_iso_(H) = 1.2*U*_eq_(C). Reflections (-2 1 2), (-2 0 4) and (0 2 4), were omitted due to the large disagreement between F_obs_ and F_calc_.

### Crystal data

**Table:**

C_28_H_27_N_3_O_5_	*F*(000) = 1024
*M**_r_* = 485.53	*D*_x_ = 1.317 Mg m^−^^3^
Monoclinic, *P*2_1_/*n*	Mo *K*α radiation, λ = 0.71073 Å
Hall symbol: -P 2yn	Cell parameters from 16797 reflections
*a* = 9.2637 (6) Å	θ = 1.8–27.6°
*b* = 11.4091 (5) Å	µ = 0.09 mm^−^^1^
*c* = 23.3310 (15) Å	*T* = 296 K
β = 96.930 (5)°	Block, light yellow
*V* = 2447.9 (2) Å^3^	0.55 × 0.38 × 0.22 mm
*Z* = 4	

### Data collection

**Table:**

Stoe IPDS 2 diffractometer	5393 independent reflections
Radiation source: sealed X-ray tube, 12 x 0.4 mm long-fine focus	3044 reflections with *I* > 2σ(*I*)
Plane graphite monochromator	*R*_int_ = 0.041
Detector resolution: 6.67 pixels mm^-1^	θ_max_ = 27.2°, θ_min_ = 1.8°
ω scans	*h* = −11→11
Absorption correction: integration (*X-RED32*; Stoe & Cie, 2002)	*k* = −14→14
*T*_min_ = 0.957, *T*_max_ = 0.983	*l* = −29→29
19036 measured reflections	

### Refinement

**Table:**

Refinement on *F*^2^	0 restraints
Least-squares matrix: full	Hydrogen site location: inferred from neighbouring sites
*R*[*F*^2^ > 2σ(*F*^2^)] = 0.041	H-atom parameters constrained
*wR*(*F*^2^) = 0.093	*w* = 1/[σ^2^(*F*_o_^2^) + (0.0457*P*)^2^] where *P* = (*F*_o_^2^ + 2*F*_c_^2^)/3
*S* = 0.88	(Δ/σ)_max_ < 0.001
5393 reflections	Δρ_max_ = 0.15 e Å^−^^3^
325 parameters	Δρ_min_ = −0.21 e Å^−^^3^

### Special details

**Table:**

Geometry. Bond distances, angles *etc*. have been calculated using the rounded fractional coordinates. All su's are estimated from the variances of the (full) variance-covariance matrix. The cell e.s.d.'s are taken into account in the estimation of distances, angles and torsion angles
Refinement. Refinement on *F*^2^ for ALL reflections except those flagged by the user for potential systematic errors. Weighted *R*-factors *wR* and all goodnesses of fit *S* are based on *F*^2^, conventional *R*-factors *R* are based on *F*, with *F* set to zero for negative *F*^2^. The observed criterion of *F*^2^ > σ(*F*^2^) is used only for calculating -*R*-factor-obs *etc*. and is not relevant to the choice of reflections for refinement. *R*-factors based on *F*^2^ are statistically about twice as large as those based on *F*, and *R*-factors based on ALL data will be even larger.

### Fractional atomic coordinates and isotropic or equivalent isotropic displacement parameters (Å^2^)

**Table:**

	*x*	*y*	*z*	*U*_iso_*/*U*_eq_	
O1	0.84675 (13)	0.41791 (9)	0.23520 (5)	0.0578 (4)	
O2	0.49859 (13)	0.68567 (11)	0.32597 (5)	0.0642 (5)	
O3	0.21884 (16)	0.55524 (13)	0.00379 (6)	0.0903 (6)	
O4	0.39138 (19)	0.44168 (14)	0.03770 (8)	0.1073 (7)	
O5	0.76395 (16)	0.93149 (14)	−0.03119 (7)	0.0914 (6)	
N1	0.80288 (13)	0.58505 (10)	0.17769 (5)	0.0425 (4)	
N2	0.33075 (18)	0.53649 (15)	0.03527 (6)	0.0641 (6)	
N3	0.86873 (15)	0.81179 (12)	0.07346 (6)	0.0539 (5)	
C1	0.78855 (17)	0.51133 (13)	0.22163 (6)	0.0424 (5)	
C2	0.67968 (16)	0.59013 (12)	0.24776 (6)	0.0406 (5)	
C3	0.69833 (16)	0.67310 (12)	0.19331 (6)	0.0405 (4)	
C4	0.74121 (18)	0.64401 (13)	0.30441 (6)	0.0447 (5)	
C5	0.88946 (19)	0.65055 (15)	0.32229 (7)	0.0566 (6)	
C6	0.9422 (2)	0.70557 (18)	0.37322 (8)	0.0727 (7)	
C7	0.8472 (3)	0.75553 (18)	0.40659 (8)	0.0746 (8)	
C8	0.6998 (2)	0.74905 (16)	0.39060 (7)	0.0667 (7)	
C9	0.64771 (19)	0.69209 (14)	0.33962 (7)	0.0512 (5)	
C10	0.44594 (19)	0.59521 (15)	0.28968 (7)	0.0533 (6)	
C11	0.3044 (2)	0.55984 (18)	0.29314 (9)	0.0704 (8)	
C12	0.2471 (2)	0.4687 (2)	0.25963 (10)	0.0783 (8)	
C13	0.3315 (2)	0.41067 (18)	0.22397 (9)	0.0729 (8)	
C14	0.4725 (2)	0.44662 (15)	0.22061 (7)	0.0588 (6)	
C15	0.53121 (18)	0.54190 (14)	0.25249 (6)	0.0465 (5)	
C16	0.57100 (16)	0.69479 (13)	0.14871 (6)	0.0405 (4)	
C17	0.50680 (17)	0.80495 (14)	0.14408 (6)	0.0484 (5)	
C18	0.39007 (18)	0.82805 (15)	0.10310 (7)	0.0557 (6)	
C19	0.33281 (18)	0.74088 (16)	0.06655 (7)	0.0535 (6)	
C20	0.39514 (17)	0.63143 (14)	0.07194 (6)	0.0462 (5)	
C21	0.51418 (17)	0.60747 (13)	0.11139 (6)	0.0454 (5)	
C22	0.89002 (19)	0.57752 (15)	0.13018 (7)	0.0534 (6)	
C23	1.0149 (2)	0.66323 (17)	0.13299 (8)	0.0672 (7)	
C24	0.9716 (2)	0.79100 (17)	0.12441 (8)	0.0637 (7)	
C25	0.8098 (3)	0.93061 (16)	0.07274 (10)	0.0799 (8)	
C26	0.7015 (3)	0.9490 (2)	0.02030 (12)	0.0950 (10)	
C27	0.8214 (2)	0.8171 (2)	−0.03121 (9)	0.0820 (8)	
C28	0.9321 (2)	0.79517 (17)	0.02015 (7)	0.0643 (7)	
H3	0.74730	0.74660	0.20560	0.0490*	
H5	0.95440	0.61730	0.29950	0.0680*	
H6	1.04170	0.70870	0.38480	0.0870*	
H7	0.88280	0.79430	0.44040	0.0900*	
H8	0.63560	0.78240	0.41360	0.0800*	
H11	0.24860	0.59770	0.31810	0.0850*	
H12	0.15120	0.44600	0.26090	0.0940*	
H13	0.29340	0.34720	0.20210	0.0870*	
H14	0.52900	0.40660	0.19670	0.0710*	
H17	0.54320	0.86430	0.16910	0.0580*	
H18	0.35000	0.90290	0.10020	0.0670*	
H19	0.25390	0.75550	0.03890	0.0640*	
H21	0.55610	0.53330	0.11300	0.0550*	
H22A	0.82710	0.59030	0.09440	0.0640*	
H22B	0.92870	0.49870	0.12900	0.0640*	
H23A	1.07620	0.64180	0.10370	0.0810*	
H23B	1.07290	0.65530	0.17030	0.0810*	
H24A	0.92910	0.81780	0.15810	0.0760*	
H24B	1.05830	0.83720	0.12160	0.0760*	
H25A	0.88840	0.98680	0.07270	0.0960*	
H25B	0.76320	0.94350	0.10730	0.0960*	
H26A	0.62100	0.89510	0.02140	0.1140*	
H26B	0.66330	1.02810	0.02080	0.1140*	
H27A	0.86620	0.80520	−0.06630	0.0980*	
H27B	0.74280	0.76090	−0.03110	0.0980*	
H28A	0.96870	0.71570	0.01860	0.0770*	
H28B	1.01330	0.84850	0.01920	0.0770*	

### Atomic displacement parameters (Å^2^)

**Table:**

	*U*^11^	*U*^22^	*U*^33^	*U*^12^	*U*^13^	*U*^23^
O1	0.0719 (8)	0.0442 (6)	0.0557 (6)	0.0105 (6)	0.0012 (6)	0.0086 (5)
O2	0.0646 (8)	0.0644 (8)	0.0658 (8)	−0.0010 (7)	0.0172 (6)	−0.0135 (6)
O3	0.0830 (10)	0.1006 (11)	0.0769 (9)	−0.0037 (8)	−0.0333 (8)	−0.0132 (8)
O4	0.1157 (13)	0.0641 (10)	0.1279 (13)	0.0029 (9)	−0.0430 (11)	−0.0246 (9)
O5	0.0794 (10)	0.0904 (11)	0.1034 (12)	0.0183 (9)	0.0075 (9)	0.0388 (9)
N1	0.0466 (7)	0.0417 (7)	0.0391 (6)	0.0052 (6)	0.0044 (6)	0.0046 (6)
N2	0.0675 (10)	0.0654 (10)	0.0551 (9)	−0.0072 (8)	−0.0104 (8)	−0.0034 (8)
N3	0.0539 (8)	0.0478 (8)	0.0602 (8)	0.0011 (7)	0.0082 (7)	0.0008 (7)
C1	0.0474 (9)	0.0388 (8)	0.0382 (8)	−0.0011 (7)	−0.0061 (7)	0.0026 (7)
C2	0.0480 (9)	0.0383 (8)	0.0347 (7)	−0.0009 (7)	0.0023 (6)	0.0033 (6)
C3	0.0447 (8)	0.0374 (8)	0.0386 (7)	−0.0003 (7)	0.0022 (6)	0.0009 (6)
C4	0.0565 (10)	0.0429 (8)	0.0339 (7)	−0.0037 (8)	0.0020 (7)	0.0043 (6)
C5	0.0568 (11)	0.0641 (11)	0.0469 (9)	−0.0088 (9)	−0.0017 (8)	0.0006 (8)
C6	0.0737 (13)	0.0866 (14)	0.0534 (10)	−0.0198 (12)	−0.0102 (10)	−0.0017 (10)
C7	0.0998 (17)	0.0782 (14)	0.0435 (10)	−0.0289 (12)	−0.0012 (11)	−0.0078 (9)
C8	0.0974 (16)	0.0619 (11)	0.0433 (9)	−0.0142 (11)	0.0190 (10)	−0.0082 (8)
C9	0.0609 (11)	0.0491 (9)	0.0438 (8)	−0.0065 (8)	0.0076 (8)	0.0029 (8)
C10	0.0532 (10)	0.0551 (10)	0.0514 (9)	−0.0036 (9)	0.0054 (8)	0.0052 (8)
C11	0.0551 (12)	0.0800 (14)	0.0777 (13)	−0.0019 (10)	0.0146 (10)	0.0084 (11)
C12	0.0570 (12)	0.0886 (15)	0.0886 (15)	−0.0207 (12)	0.0055 (11)	0.0157 (13)
C13	0.0721 (14)	0.0705 (13)	0.0738 (13)	−0.0266 (11)	0.0000 (11)	0.0004 (11)
C14	0.0647 (12)	0.0560 (10)	0.0551 (10)	−0.0142 (9)	0.0045 (9)	−0.0001 (8)
C15	0.0517 (10)	0.0458 (9)	0.0407 (8)	−0.0045 (8)	0.0009 (7)	0.0075 (7)
C16	0.0445 (8)	0.0419 (8)	0.0353 (7)	0.0014 (7)	0.0053 (6)	0.0033 (7)
C17	0.0565 (10)	0.0451 (9)	0.0433 (8)	0.0069 (8)	0.0045 (8)	−0.0021 (7)
C18	0.0584 (11)	0.0520 (10)	0.0556 (10)	0.0184 (8)	0.0020 (8)	0.0012 (8)
C19	0.0456 (9)	0.0694 (11)	0.0444 (9)	0.0093 (9)	0.0005 (8)	0.0059 (8)
C20	0.0476 (9)	0.0521 (9)	0.0385 (8)	−0.0022 (8)	0.0031 (7)	0.0006 (7)
C21	0.0517 (9)	0.0416 (9)	0.0420 (8)	0.0032 (7)	0.0015 (7)	0.0038 (7)
C22	0.0622 (11)	0.0526 (10)	0.0472 (9)	0.0087 (9)	0.0145 (8)	0.0023 (8)
C23	0.0517 (10)	0.0897 (15)	0.0603 (11)	0.0010 (10)	0.0072 (9)	0.0204 (10)
C24	0.0626 (12)	0.0716 (12)	0.0571 (10)	−0.0203 (10)	0.0085 (9)	0.0010 (9)
C25	0.0907 (15)	0.0502 (11)	0.1043 (16)	0.0055 (11)	0.0337 (14)	−0.0014 (11)
C26	0.0768 (15)	0.0736 (15)	0.136 (2)	0.0262 (12)	0.0188 (16)	0.0281 (15)
C27	0.0839 (15)	0.0882 (16)	0.0705 (13)	0.0121 (12)	−0.0048 (11)	0.0079 (12)
C28	0.0641 (12)	0.0697 (12)	0.0586 (10)	0.0146 (10)	0.0059 (9)	0.0062 (9)

### Geometric parameters (Å, º)

**Table:**

O1—C1	1.2189 (18)	C18—C19	1.374 (2)
O2—C9	1.382 (2)	C19—C20	1.375 (2)
O2—C10	1.386 (2)	C20—C21	1.376 (2)
O3—N2	1.215 (2)	C22—C23	1.510 (3)
O4—N2	1.217 (2)	C23—C24	1.519 (3)
O5—C26	1.409 (3)	C25—C26	1.500 (4)
O5—C27	1.410 (3)	C27—C28	1.501 (3)
N1—C1	1.3449 (18)	C3—H3	0.9800
N1—C3	1.4715 (19)	C5—H5	0.9300
N1—C22	1.450 (2)	C6—H6	0.9300
N2—C20	1.462 (2)	C7—H7	0.9300
N3—C24	1.451 (2)	C8—H8	0.9300
N3—C25	1.461 (2)	C11—H11	0.9300
N3—C28	1.450 (2)	C12—H12	0.9300
C1—C2	1.532 (2)	C13—H13	0.9300
C2—C3	1.610 (2)	C14—H14	0.9300
C2—C4	1.506 (2)	C17—H17	0.9300
C2—C15	1.498 (2)	C18—H18	0.9300
C3—C16	1.496 (2)	C19—H19	0.9300
C4—C5	1.388 (2)	C21—H21	0.9300
C4—C9	1.378 (2)	C22—H22A	0.9700
C5—C6	1.380 (3)	C22—H22B	0.9700
C6—C7	1.368 (3)	C23—H23A	0.9700
C7—C8	1.373 (3)	C23—H23B	0.9700
C8—C9	1.390 (2)	C24—H24A	0.9700
C10—C11	1.384 (3)	C24—H24B	0.9700
C10—C15	1.383 (2)	C25—H25A	0.9700
C11—C12	1.369 (3)	C25—H25B	0.9700
C12—C13	1.378 (3)	C26—H26A	0.9700
C13—C14	1.380 (3)	C26—H26B	0.9700
C14—C15	1.390 (2)	C27—H27A	0.9700
C16—C17	1.389 (2)	C27—H27B	0.9700
C16—C21	1.385 (2)	C28—H28A	0.9700
C17—C18	1.380 (2)	C28—H28B	0.9700
			
C9—O2—C10	116.66 (13)	C2—C3—H3	111.00
C26—O5—C27	108.99 (17)	C16—C3—H3	111.00
C1—N1—C3	96.30 (11)	C4—C5—H5	119.00
C1—N1—C22	131.36 (13)	C6—C5—H5	119.00
C3—N1—C22	132.33 (12)	C5—C6—H6	120.00
O3—N2—O4	122.68 (17)	C7—C6—H6	120.00
O3—N2—C20	118.78 (16)	C6—C7—H7	120.00
O4—N2—C20	118.54 (16)	C8—C7—H7	120.00
C24—N3—C25	111.64 (15)	C7—C8—H8	120.00
C24—N3—C28	112.80 (14)	C9—C8—H8	120.00
C25—N3—C28	107.51 (15)	C10—C11—H11	120.00
O1—C1—N1	131.57 (14)	C12—C11—H11	120.00
O1—C1—C2	134.57 (14)	C11—C12—H12	120.00
N1—C1—C2	93.82 (11)	C13—C12—H12	120.00
C1—C2—C3	83.83 (10)	C12—C13—H13	120.00
C1—C2—C4	113.04 (12)	C14—C13—H13	120.00
C1—C2—C15	117.93 (12)	C13—C14—H14	120.00
C3—C2—C4	112.78 (11)	C15—C14—H14	120.00
C3—C2—C15	117.45 (12)	C16—C17—H17	119.00
C4—C2—C15	109.82 (12)	C18—C17—H17	119.00
N1—C3—C2	86.02 (10)	C17—C18—H18	120.00
N1—C3—C16	115.33 (12)	C19—C18—H18	120.00
C2—C3—C16	119.69 (12)	C18—C19—H19	121.00
C2—C4—C5	122.79 (14)	C20—C19—H19	121.00
C2—C4—C9	119.16 (15)	C16—C21—H21	120.00
C5—C4—C9	118.02 (14)	C20—C21—H21	120.00
C4—C5—C6	121.18 (16)	N1—C22—H22A	108.00
C5—C6—C7	119.63 (18)	N1—C22—H22B	109.00
C6—C7—C8	120.65 (18)	C23—C22—H22A	109.00
C7—C8—C9	119.26 (17)	C23—C22—H22B	109.00
O2—C9—C4	121.64 (14)	H22A—C22—H22B	108.00
O2—C9—C8	117.15 (15)	C22—C23—H23A	108.00
C4—C9—C8	121.21 (16)	C22—C23—H23B	108.00
O2—C10—C11	116.60 (16)	C24—C23—H23A	108.00
O2—C10—C15	121.69 (15)	C24—C23—H23B	108.00
C11—C10—C15	121.70 (16)	H23A—C23—H23B	107.00
C10—C11—C12	119.55 (18)	N3—C24—H24A	109.00
C11—C12—C13	120.04 (18)	N3—C24—H24B	109.00
C12—C13—C14	120.09 (18)	C23—C24—H24A	109.00
C13—C14—C15	120.91 (17)	C23—C24—H24B	109.00
C2—C15—C10	118.93 (14)	H24A—C24—H24B	108.00
C2—C15—C14	123.45 (14)	N3—C25—H25A	109.00
C10—C15—C14	117.61 (16)	N3—C25—H25B	110.00
C3—C16—C17	119.86 (13)	C26—C25—H25A	110.00
C3—C16—C21	121.75 (13)	C26—C25—H25B	110.00
C17—C16—C21	118.39 (13)	H25A—C25—H25B	108.00
C16—C17—C18	121.28 (14)	O5—C26—H26A	109.00
C17—C18—C19	120.22 (16)	O5—C26—H26B	109.00
C18—C19—C20	118.30 (15)	C25—C26—H26A	109.00
N2—C20—C19	118.82 (14)	C25—C26—H26B	109.00
N2—C20—C21	118.80 (14)	H26A—C26—H26B	108.00
C19—C20—C21	122.37 (14)	O5—C27—H27A	109.00
C16—C21—C20	119.40 (14)	O5—C27—H27B	109.00
N1—C22—C23	115.00 (14)	C28—C27—H27A	109.00
C22—C23—C24	115.28 (15)	C28—C27—H27B	109.00
N3—C24—C23	113.70 (15)	H27A—C27—H27B	108.00
N3—C25—C26	110.57 (17)	N3—C28—H28A	109.00
O5—C26—C25	111.9 (2)	N3—C28—H28B	109.00
O5—C27—C28	112.07 (17)	C27—C28—H28A	110.00
N3—C28—C27	110.76 (15)	C27—C28—H28B	110.00
N1—C3—H3	111.00	H28A—C28—H28B	108.00
			
C9—O2—C10—C11	155.89 (16)	C1—C2—C15—C14	−19.2 (2)
C10—O2—C9—C4	23.8 (2)	C15—C2—C3—N1	−119.59 (13)
C10—O2—C9—C8	−156.04 (15)	C1—C2—C15—C10	161.87 (14)
C9—O2—C10—C15	−23.2 (2)	C3—C2—C15—C14	78.76 (19)
C27—O5—C26—C25	57.9 (2)	C1—C2—C4—C9	−163.90 (14)
C26—O5—C27—C28	−57.7 (2)	C2—C3—C16—C17	110.27 (15)
C1—N1—C22—C23	−110.50 (18)	C2—C3—C16—C21	−69.70 (18)
C3—N1—C1—O1	−179.56 (17)	N1—C3—C16—C21	30.8 (2)
C22—N1—C1—C2	179.91 (15)	N1—C3—C16—C17	−149.28 (13)
C22—N1—C1—O1	1.9 (3)	C5—C4—C9—O2	−177.71 (14)
C3—N1—C1—C2	−1.50 (12)	C9—C4—C5—C6	−1.3 (2)
C3—N1—C22—C23	71.4 (2)	C2—C4—C5—C6	176.86 (16)
C1—N1—C3—C16	−119.66 (13)	C2—C4—C9—O2	4.1 (2)
C22—N1—C3—C2	180.00 (15)	C5—C4—C9—C8	2.2 (2)
C22—N1—C3—C16	58.9 (2)	C2—C4—C9—C8	−176.06 (15)
C1—N1—C3—C2	1.43 (11)	C4—C5—C6—C7	−0.6 (3)
O3—N2—C20—C21	174.10 (15)	C5—C6—C7—C8	1.6 (3)
O4—N2—C20—C19	175.60 (17)	C6—C7—C8—C9	−0.7 (3)
O4—N2—C20—C21	−5.7 (2)	C7—C8—C9—O2	178.67 (16)
O3—N2—C20—C19	−4.6 (2)	C7—C8—C9—C4	−1.2 (3)
C24—N3—C25—C26	−178.81 (18)	C11—C10—C15—C14	−3.4 (2)
C28—N3—C24—C23	−69.47 (19)	O2—C10—C15—C2	−5.3 (2)
C25—N3—C24—C23	169.34 (17)	C11—C10—C15—C2	175.60 (16)
C28—N3—C25—C26	57.0 (2)	C15—C10—C11—C12	0.9 (3)
C25—N3—C28—C27	−56.8 (2)	O2—C10—C11—C12	−178.17 (18)
C24—N3—C28—C27	179.73 (16)	O2—C10—C15—C14	175.66 (15)
O1—C1—C2—C15	−62.8 (2)	C10—C11—C12—C13	1.8 (3)
O1—C1—C2—C3	179.33 (19)	C11—C12—C13—C14	−2.0 (3)
O1—C1—C2—C4	67.2 (2)	C12—C13—C14—C15	−0.6 (3)
N1—C1—C2—C15	119.25 (13)	C13—C14—C15—C10	3.2 (2)
N1—C1—C2—C4	−110.79 (13)	C13—C14—C15—C2	−175.76 (16)
N1—C1—C2—C3	1.37 (11)	C21—C16—C17—C18	−0.6 (2)
C1—C2—C4—C5	18.0 (2)	C3—C16—C17—C18	179.44 (14)
C15—C2—C3—C16	−2.59 (19)	C3—C16—C21—C20	178.70 (14)
C4—C2—C15—C14	−150.62 (15)	C17—C16—C21—C20	−1.3 (2)
C15—C2—C4—C9	−29.94 (19)	C16—C17—C18—C19	1.4 (2)
C3—C2—C4—C9	103.12 (16)	C17—C18—C19—C20	−0.2 (2)
C15—C2—C4—C5	151.94 (15)	C18—C19—C20—C21	−1.7 (2)
C4—C2—C3—C16	−131.83 (14)	C18—C19—C20—N2	176.99 (15)
C3—C2—C15—C10	−100.18 (16)	C19—C20—C21—C16	2.5 (2)
C4—C2—C3—N1	111.17 (13)	N2—C20—C21—C16	−176.22 (14)
C1—C2—C3—C16	115.75 (13)	N1—C22—C23—C24	−68.6 (2)
C1—C2—C3—N1	−1.25 (10)	C22—C23—C24—N3	−50.6 (2)
C4—C2—C15—C10	30.44 (19)	N3—C25—C26—O5	−59.3 (2)
C3—C2—C4—C5	−75.00 (18)	O5—C27—C28—N3	59.0 (2)

### Hydrogen-bond geometry (Å, º)

**Table:**

*D*—H···*A*	*D*—H	H···*A*	*D*···*A*	*D*—H···*A*
C17—H17···O1^i^	0.93	2.42	3.2423 (19)	148
C22—H22*A*···N3	0.97	2.61	2.978 (2)	103

Symmetry code: (i) −*x*+3/2, *y*+1/2, −*z*+1/2.
